# Access to early diagnosis for attention-deficit/hyperactivity disorder among children and adolescents in Mexico City at specialized mental health services

**DOI:** 10.1186/s12913-024-11022-y

**Published:** 2024-05-07

**Authors:** María Magdalena Martínez-Jaime, Hortensia Reyes-Morales, Ixchel Peyrot-Negrete, Mauricio Sebastián Barrientos-Álvarez

**Affiliations:** 1 Hospital Psiquiátrico Infantil “Dr. Juan N. Navarro”, San Buenaventura 86. Col. Belisario Domínguez Tlalpan, México City, 14080 México; 2 del Centro de Investigación en Sistemas de Salud, Instituto Nacional de Salud Pública, Avenida Universidad 655, Santa María Ahuacatitlán, Cuernavaca, Morelos 62100 México; 3 Universidad Nacional Autonóma de México, Av. Universidad 3000, Alcaldía Coyoacan Ciudad de Mexico, C.P. 04510 México City, México; 4 Universidad del Desarrollo, Av. Plaza 680. Las Condes, Santiago, Chile

**Keywords:** Attention-deficit/hyperactivity disorder (ADHD), Access, Timely diagnosis, Delays

## Abstract

**Background:**

In Mexico, this pioneering research was undertaken to assess the accessibility of timely diagnosis of Dyads [Children and adolescents with Attention Deficit Hyperactivity Disorder (ADHD) and their primary caregivers] at specialized mental health services. The study was conducted in two phases. The first phase involved designing an “Access Pathway” aimed to identify barriers and facilitators for ADHD diagnosis; several barriers, with only the teacher being identified as a facilitator. In the second phase, the study aimed to determine the time taken for dyads, to obtain a timely diagnosis at each stage of the Access Pathway. As well as identify any disparities based on gender and socioeconomic factors that might affect the age at which children can access a timely diagnosis.

**Method:**

In a retrospective cohort study, 177 dyads participated. To collect data, the Acceda Survey was used, based on the robust Conceptual Model Levesque, 2013. The survey consisted of 48 questions that were both dichotomous and polytomous allowing the creation of an Access Pathway that included five stages: the age of perception, the age of search, the age of first contact with a mental health professional, the age of arrival at the host hospital, and the age of diagnosis. The data was meticulously analyzed using a comprehensive descriptive approach and a nonparametric multivariate approach by sex, followed by post-hoc Mann-Whitney’s U tests. Demographic factors were evaluated using univariable and multivariable Cox regression analyses.

**Results:**

71% of dyads experienced a late, significantly late, or highly late diagnosis of ADHD. Girls were detected one year later than boys. Both boys and girls took a year to seek specialized mental health care and an additional year to receive a formal specialized diagnosis. Children with more siblings had longer delays in diagnosis, while caregivers with formal employment were found to help obtain timely diagnoses.

**Conclusions:**

Our findings suggest starting the Access Pathway where signs and symptoms of ADHD are detected, particularly at school, to prevent children from suffering consequences. Mental health school-based service models have been successfully tested in other latitudes, making them a viable option to shorten the time to obtain a timely diagnosis.

## Background

Attention-deficit/hyperactivity disorder (ADHD) is a neurodevelopmental disorder characterized by a persistent pattern of inattention, hyperactivity, and impulsivity that is pervasive across settings and leads to various degrees of functional impairment [1]. ADHD strongly impacts individuals’ functions; this is worsened when individuals are undiagnosed, and risks such as increased imprisonment, depression, or drug misuse are often observed; if left undiagnosed and untreated, ADHD can cause a significant economic burden on society [2].

According to European [3, 4], American [1], and Latin American consensus [5], an early diagnosis is necessary to propose better and earlier psychoeducational interventions that agree with a preventive perspective. The Association of Academy of Pediatrics (AAP) played a key role in this initiative by forming a subcommittee on ADHD, overseen by the AAP Council on Quality Improvement and Patient Safety. The Key Action Statement (KAS) highly recommends initiating an evaluation for ADHD for any child or adolescent aged four years to the 18th birthday who presents academic or behavioral problems and symptoms of inattention, hyperactivity, or impulsivity according to DSM-V [1]. From the perspective of the European ADHD Guidelines Group, treatment recommendations vary by age, with preschoolers receiving behavioral interventions as the first line and older children prescribed FDA-approved medications alongside behavioral interventions [3, 4].

ADHD is a disorder that affects children and adolescents from an early age. It is linked to other disorders that can last into adulthood [6]. Recent research has found that the time gap between age of onset and diagnosis varies widely across different European countries [7]. This large gap highlights the importance of early intervention, which can help people with ADHD reach their full potential and reduce the negative impact of the disorder. In many countries, including Mexico, children and adolescents experience delays in accessing mental health services care, which can worsen their condition.

It is essential to highlight that both the Diagnostic and Statistical Manual of Mental Disorders (DSM-5) and the International Classification of Diseases (ICD-11) [8, 9] only mention that when notice the presence of core symptoms of ADHD or Age of Onset should be present before the age of 12. However, due to the high variability of diagnostic criteria, the age for an early diagnosis is often unclear. When developing the ICF Core Sets for ADHD, it is essential to consider cultural and attitudinal differences [10] because symptoms of neurodevelopmental disorders may appear similar across cultures [11]. However, these studies did not examine the differences in ADHD diagnosis time between the interpretation, perception, and acceptance of these symptoms vary greatly [12].

Then, some experts stand out that the heterogeneity in the methodology of diagnosing ADHD has resulted in high variability in prevalence rates around the world, and they affirm that it needs better clarification. Moreover, differences linked to Age of Onset and the Age of Diagnosis of ADHD require further investigation [13]. It is necessary to note the dependence of clinicians on diagnostic manuals such as the DSM5, the ICD-11, and the Research Domain Criteria. So, it would be necessary to have a standard set of criteria for attitude and practice when requesting an appointment.

The Fridman [14] and Bonati [12] research groups conclude that the essential intervention to reduce differences between centers in access should be the definition and utilization of common criteria and practice since the first request for an appointment, at the same time both identified gaps in access to diagnosis and supportive care, highlighting the need for improved access to diagnosis and supportive services and mentioning that there is a need for better communication between parents, schools, healthcare providers, and their administrative services, to alleviate the burden on parents, comprehensive and standardized professional training and guidance for teachers and parents may be helpful. Fridman concluded that support for schools in many European countries is a fact, with approximately 15% attending a school for special needs and 2% being home-schooled or attending some other type of school, demonstrating significant variability between countries (*p* < .001).

Symptomatic presentation can lead to underdiagnosing and identifying girls with ADHD [15, 16]. . Nevertheless, these two previous studies [12, 14] estimated prolonged times to obtain an ADHD diagnosis but did not examine the differences between boys and girls. Despite that, some authors distinguished considerable differences, suggesting sex inequalities in access to a timely diagnosis [17, 18]. These disparities must be considered, including the differences in the symptomatic presentation, as they lead to girls with ADHD being unidentified and underdiagnosed [16].

The only study conducted by Caraveo, in Mexico City to investigate the symptoms of ADHD in children and adolescents aged 4 to 16 years. The study aimed to find out how caregivers detect symptoms and seek help to determine the disorder’s presence or development. Caraveo’s results are in agreement with ours since he also highlights the lack of recognition of ADHD symptoms as potential mental health problems. He mentions in his discussion that his findings indicate a lack of awareness regarding the significance of some psychopathological manifestations that occur during childhood and adolescence. It is concerning that the critical symptoms of attention deficit hyperactivity disorder, which are among the most prevalent manifestations and also appear early in life, have not been recognized as reasons for concern or as a cause for minors to seek medical attention. Only poor academic performance (a consequence of poor attention but not exclusively related to it) was considered an issue. The author agrees that less than half of the symptomatic minors received care, with seeking of care being even lower at just 13% [19].

Without any doubt, there is a significant shortage of specialized mental health services in Mexico City, with only one Children’s Psychiatric Hospital. Considering that there are 2,300,000 girls and boys aged 0 to 19 years in Mexico City [20], , this raises concerns about the availability of mental health services. The Children’s Psychiatric Hospital is the only facility that offers a formal, interdisciplinary, and evidence-based diagnosis, but it has minimal availability, which limits access to mental health services for the population without age limits.

This brode research aimed to evaluate the accessibility of mental health services that are tailored to specific needs, profiting Levesque’s conceptual model [21]; utilizing the same sample for data collection. The first part of this research examined variables at a descriptive level to identify the barriers and facilities present in the Access Pathway. The Access Pathway comprises five stages involved in obtaining a diagnosis, including the age of perception, age of search, age of first contact with a mental health professional, age of arrival at a hospital, and age of diagnosis. The study found that all caregivers faced difficulty in identifying core symptoms and that specialized care was inaccessible due to the distance of the hospital from their homes. Additionally, 95% of caregivers experienced long wait times for diagnosis and treatment without any apparent decrease in symptoms. These variables were categorized as barriers, with only one facilitator identified - the school sector. Teachers were found to detect symptoms and urge caregivers to seek help. This could help clinicians and policymakers understand the factors associated with delays in diagnosis and optimize specialized services processes [22].

In this other part of the research, we analyzed variables that were not examined in a previously published study. Our main objectives and the variables analyzed were different from those in the previous study. Specifically, we aimed to determine the time it takes between the age of perception and the age of diagnosis, the time it takes between each dimension of the Access Pathway, and to identify any differences between males and females. Additionally, we examined the sociodemographic factors that cause delays in obtaining a diagnosis. It was essential to establish an appropriate age for diagnosis to prevent significant consequences on the mental health of children and adolescents.

## Methods

This is a retrospective cohort study that was conducted in the Mexican public health sector’s only tertiary hospital for children and adolescents. A total of 177 dyads (child or adolescent with ADHD and their primary caregiver) participated.

**Fig. 1:**
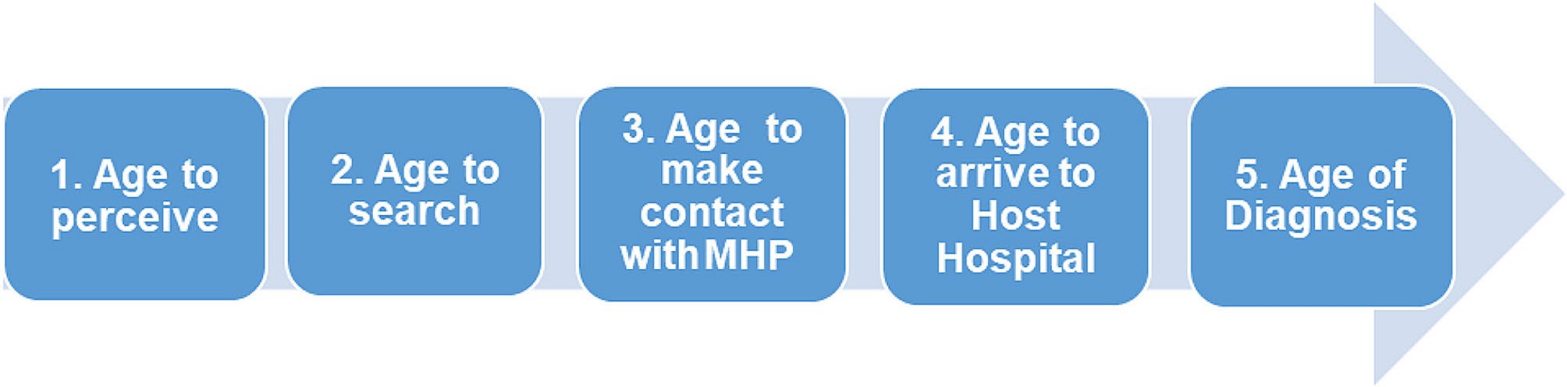
Access pathway to diagnosis. Note: MHP: Mental Health Professional; HH: host hospital

An Access Pathway to diagnosis, based on the Levesque Conceptual Model [21], was used as the basis for the analysis because it allowed us to place the dyads at the center of the process and investigate their abilities to interact with health services. The pathway has five phases, representing the pathway to the diagnosis, based on the child’s age: (1) age of perception of the problem, (2) age of search for a mental health professional (MHP), (3) age of first visit to the MHP, (4) age of reference to specialized diagnostic services in the host hospital (HH), and (5) age of diagnosis. The Access Pathway was used to identify barriers to timely diagnosis for children with ADHD by asking questions to the primary caregivers (PCs) as shown in Fig. 1.

Additionally, two measures were established: (a) the appropriate age for diagnosis was determined to be four years old, based on the impact of ADHD on the child, family, and community from preschool to adulthood, and (b) the ideal timeframe for completing the pathway from initial recognition to diagnosis was estimated in six months. Therefore, the optimal age for diagnosis was considered to be ≤ 4.5 years [2].

### The AAcceDa survey

From Levesque’s Model, and in order to evaluate the Access Pathway, a semi-structured questionnaire called the AAcceDa survey (Acceptability, Accessibility, Availability, and Approach) was designed to collect data regarding access to timely care for ADHD. To track the Access Pathway, the age of each child was recorded at five different stages, along with the length of time between each stage. The questions included who first noticed the child’s changing behavior, who decided to seek help, who suggested seeking help, and so on.

The questionnaire is composed of 48 mixed questions: five open-ended and 43 dichotomous or polytomous questions aimed to gather information regarding the children’s behavior, and access to diagnosis and treatment, as well as sociodemographic information about the children and their families. This survey allowed us to identify and track the five phases along the pathway based on the child’s age. The evaluation process was dynamic and continuous since each phase influenced the others at different times during the diagnosis and care of the disorder.

### AAcceDa survey validation and piloting

The AcceDa survey was validated through a face validity method, which assesses the clarity and understanding of the questionnaire; the survey underwent a validation and piloting process in order to improve it for better efficiency. The procedure of validation consisted of three steps. In the first step, two meetings were held by three specialists on ADHD, mental health interventions, and health systems to analyze the proposed questions. In these meetings, they considered the relevance of every topic and item. In the second step, an expert in instrument design improved question clarity and checked the appropriateness of every question. Finally, social workers and pediatric psychiatrists evaluated whether the questionnaire was user-friendly or not.

The piloting procedure was equally strict; it consisted of three rounds of testing. Initially, three social workers organized a psychoeducational workshop for parents of minors with ADHD to evaluate the questionnaire’s suitability for self-administration. Subsequently, twelve dyads not included in the main study participated in the second round of piloting, providing valuable feedback. In the third round, the lead researcher, along with the instrument design expert and pediatric psychiatrists, thoroughly reviewed and discussed the entire survey to assess its relevance and wording.

The results of the face validity assessment showed that the survey needed modifications. Initially comprising 75 questions, 12 questions related to Stigma and Grief were deemed as less essential and eliminated from the list. Additionally, questions regarding financial challenges in meeting care, medication, and transportation costs were reworded and streamlined, resulting in the elimination of further eight questions. Finally, feedback from social workers emphasized the importance of visual aids to foster understandability. The pilot results showed that, despite the survey’s generally favorable reception, there were issues regarding the limited feasibility for self-application due to recall bias. To address this, interviews were conducted with the pilot’s participants to mitigate bias, yielding valuable insights. The final version of the questionnaire was reviewed and approved by experts in order to ensure its relevance and clarity in relation to ADHD and parental experiences research.

### Statistical analysis

The data was analyzed through a descriptive approach, which presented the data as numbers, percentages, means, standard deviation, medians, and interquartile ranges. The assumption of multivariate normality required for the MANOVA analysis was not met (*R* = 97.60, *p* < .001), so a nonparametric multivariate inferential approach was used to compare data between sexes. For this comparative analysis, the “npmv” R package was used, which allowed a multivariate global test of statistical significance to be calculated, followed by post-hoc univariate Mann-Whitney’s U tests. Demographic factors related to age diagnosis were evaluated through univariable and multivariable Cox regression analyses. A statistically significant p-value < 0.05 was considered. The analyses were conducted using the STATA 14 statistical software (Stata Corp, College Station, TX, USA) and R v.4.3.1 (R Core Team, Austria).

## Results

### Sociodemographic characteristics of dyads

A total of 177 dyads were examined, with the average age of the child being 8.2 years at the time of diagnosis. Boys made up the majority of the group, accounting for 81.4% of the sample. The diagnosis was found to be 5.3 times more frequent in boys than in girls. About 60% of the dyads were affiliated with Mexican public health insurance (Seguro Popular), while 20% had social security, and 20% had no health insurance. In terms of school performance, 15 children repeated the same grade, and it was the primary caregivers (PCs) who requested this repetition due to concerns about their children’s poor academic performance. The ages of the primary caregivers ranged from 13 to 72 years, with a median age of 40 years. The majority of the primary caregivers were mothers, accounting for 85% of the sample, while 7% were grandmothers and 3% were aunts or sisters. Additionally, 43% of the primary caregivers declared that they were solely responsible for raising and caring for the child. For more information on the sociodemographic data of the primary caregivers, please refer to Table 1.

**Table 1:** Descriptive demographics of primary caregivers (PCs)

Age	Median	Range
	40	13–72
Sex	n	%
Men	10	5.65
Women	167	94.35
Education level		
1st to 3rd primary school year	11	6.21
4th to 6th primary school year	17	9.6
Elementary Middle school	63	35.59
High school	55	31.07
College	31	17.51
Relationship with child		
Mother	150	84.75
Father	8	4.52
Grandparents	13	7.34
Aunts, sisters	6	3.39
Occupation		
Housewife	90	50.85
Formal employment	34	19.21
Informal employment	53	29.94
Health insurance		
Partial health insurance	105	59.32
Total health insurance	36	20.34
None	36	20.34

### Delays’ access to an early diagnosis

### (Children’s ages at each phase of the pathway)

Both boys and girls faced significant delays in obtaining a timely diagnosis. On average, it took a year for both genders to receive a formal diagnosis. One of the primary reasons for the delay was that primary caregivers often did not recognize the core symptoms as mental health problems. As a result, teachers were usually the first to identify the problem and ask dyads to seek help. However, teachers tend to recognize externalized symptoms more often in boys than internalized symptoms presented by girls, leading to a higher rate of diagnosis for boys (Table 2).

The difficulty in obtaining a diagnosis within the optimal time was evident, considering the substantial delays that both boys and girls experienced across the five phases in the whole pathway. The comparison of median times showed that both boys and girls waited for one year to obtain a formal diagnosis. Due to the lack of awareness among primary caregivers about core symptoms being mental health problems, teachers are the primary ones to identify the problem and request care. Since teachers tend to perceive internalized symptoms presented by girls less frequently than the externalized symptoms in boys, boys end up being diagnosed more frequently than girls (Table 2).

Delays in access to complete the whole pathway for boys and girls resulted in enormous difficulty in obtaining a formal diagnosis, as revealed by the wide variability in the ages of diagnosis, ranging from 4.46 to 15.25 years for boys, with an interquartile range between 6.26 and 9.1 years. Girls also showed remarkable delays and even more variability in the diagnosis age range than boys; this was between 6.11 and 17.1 years, with an interquartile range between 7.17 and 9.41 years. According to the median age, girls began their pathway to diagnosis one year and a half years later than boys. These results are shown in Table 2.

The difference in ages between boys and girls persisted throughout the whole pathway; the median age at which caregivers perceived the problem was five years in boys and six years in girls. The median age at which boys were diagnosed was 7.33 years, while the median age at diagnosis for girls was 8.33 years. Times to cross the complete pathway did not obey a pattern. Variability was the rule in boys and girls and any individual regarding the age of perception, seeking MHP, arriving at the HH, and being diagnosed presented a different pace of time in obtaining a formal diagnosisat diferent age (Table 2).

**Table 2:** Displays the ages of the children in whole phases of the access pathway

	Age (years)					
*Phase*	**Boys (** ***n*** ** = 144)**			**Girls (** ***n*** ** = 33)**		
	**Median**	**Range**	**IQR**	**Median**	**Range**	**IQR**
1 Age First perception	5	0.5–12	3	6	1–16	2
2 Age Search for MHP	6	3–13	2	7	3–16	1
3 First Contact with MHP	6.5	3.33–13.5	2	7.08	4–16	2.75
4 Visit HH	7.17	4.42–15	2.83	8	6–17	2.08
5 Age of Diagnosis	7.33	4.46–15.25	2.82	8.33	6.11–17.1	2.24

Note: IQR: interquartile range; MHP: mental health professional; HH: host hospital

### The time it takes to diagnose extreme age-related delays compared to the optimal age

Indicators were developed to identify the best age for diagnosing minors with core symptoms, based on research that describes these symptoms from an early age. These indicators were then used to evaluate any delays in diagnosis based on the age of the minors. Unfortunately, only one boy was diagnosed at the optimal age, and none of the girls were. Optimal diagnosed at or before 4.5 years of age) Less than one-third of the participants received a timely diagnosis (i.e., diagnosed at or before 6.5 years of age). It’s unacceptable that only one boy was diagnosed at the optimal age of 4.5 years, and none of the girls were. The fact that less than one-third of the participants received a timely diagnosis, that is, before the age of 6.5 years, clearly indicates the need for urgent action to improve the diagnostic process.

The majority of the children were diagnosed late, with approximately half of them receiving a diagnosis in their last three years of primary school (as illustrated in Table 3). When the delayed indicators of diagnosis were taken into account, it was found that 71% of the participants received their diagnosis at a late, considerably late, or extremely late age (as shown in Table 3). The percentage of boys diagnosed at an appropriate age was more than double that of girls (31.25% vs. 12.12%). Nevertheless, it was more common for both groups to receive a diagnosis at a late age.

**Table 3:** Indicators based on optimal age of diagnosis (frequencies by sex)

Indicators of time opportunity	Boys		Girls		Total	
	**n**	%	**n**	%	**n**	%
Optimal diagnostic age (≤ 4.5 years)	1	0.69	0	0	1	0.56
Timely diagnostic age (4.5–6.5 years)	44	30.56	4	12.12	48	27.12
Late diagnostic age (6.5–8.5 years)	51	35.42	14	42.42	65	36.72
Considerably late diagnostic age (8.5–11.5 years)	35	24.31	10	30.3	45	25.42
Excessively late diagnostic age (11.5–17.5 years)	13	9.03	5	15.15	18	10.17
Total	144	100	33	100	177	100

### Length of each time interval

**Fig. 2:**
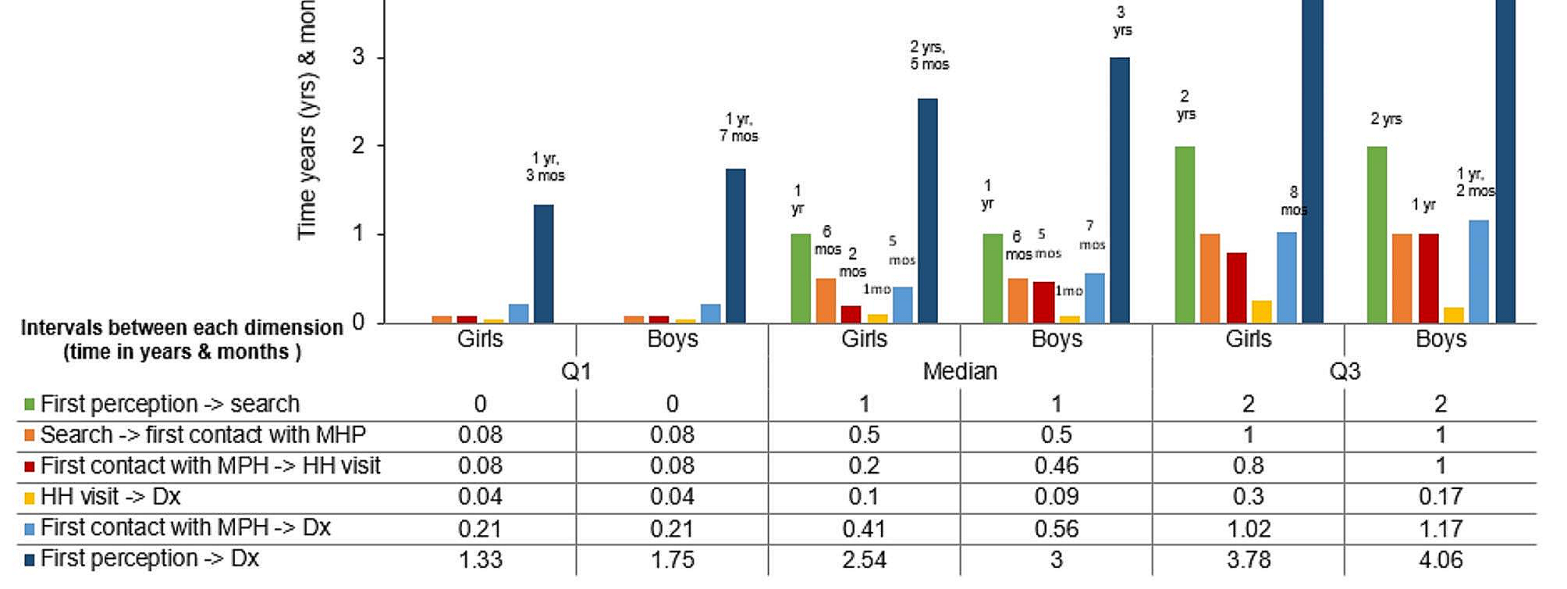
Quartiles of time intervals for children of different ages through an access pathway (medians)

Based on the medians presented in Fig. 2, the longest delay in obtaining an ADHD diagnosis occurs during the time from recognizing the problem to seeking help from a mental health professional (MHP). The delay lasts for an average of one year for both boys and girls. This delay might occur because primary caregivers (PC) are unaware of the core symptoms of ADHD, which include inattention, hyperactivity, and impulsivity, as potential mental health issues. As a result, caregivers do not seek care as they do not perceive ADHD symptoms as potential mental health problems. This leads to significant delays in accessing specialized mental health services. According to the medians shown in Fig. 2, the pathway to diagnosis for boys’ dyads starts at the age of five, while for girls, it starts at the age of six.

It was found that boys began accessing the pathway for ADHD diagnosis a year earlier than girls. However, it took boys’ dyads three months longer than girls’ dyads to complete the pathway, implying that boys faced more difficulties in crossing the whole Access Pathway. The most significant obstacles were the lack of a reference and counter-reference system for children requiring ADHD care, and the retention of psychologists in the therapeutic process without addressing their care needs or referring them to the Host Hospital for treatment. There were notable differences in the time taken to receive a formal diagnosis, regardless of gender, across all quartiles, as shown in Fig. 2.

### Delay´s differences between boys and girls

Our research aimed to investigate whether there are differences in ADHD diagnosis delays between boys and girls. We conducted a nonparametric multivariate analysis that showed a statistically significant global difference between the two sexes [F(1.83, 191.62) = 5.09, *p* = .009]. To further assess the differences, we used Mann-Whitney U tests for each phase of the access pathway. Our analysis revealed statistically significant differences in practically all the pathway phases, except for the age of the first perception of the problem. For more information and details, please refer to Table 4; Fig. 2.

**Table 4:** Sex differences according to the age of minors, through the access pathway

Phase	Age (median)		*p*-value
	Boys	Girls	
The first perception of the problem	5	6	0.092
Search for MHP	6	7	0.045*
First visit with MHP	6.5	7.08	0.033*
Hospital visit	7.17	8	0.030*
Diagnosis	7.33	8.33	0.031*

### Univariable and multivariable Cox regression analyses

We conducted a series of univariable Cox regression analyses to determine which sociodemographic factors affect the age at which ADHD is diagnosed. Based on the findings, the child’s gender, number of siblings, PC age, and PC occupation were selected as variables to include in a multivariable model (refer to Table 5 for more details). We then conducted a multivariable Cox regression with backward stepwise elimination (α = 0.10) and found that child gender, number of siblings, and PC occupation were associated with the age at which ADHD is diagnosed. These results showed that timely ADHD diagnoses were less likely for girls (Adjusted Hazard Ratio = 0.57, 95% CI: 0.37–0.86), children with siblings (Adjusted Hazard Ratio = 0.63, 95% CI: 0.44–0.91), and more likely for children whose PC had formal employment (Adjusted Hazard Ratio = 1.45, 95% CI: 1.00-2.11). These findings emphasize the importance of considering these demographic factors when diagnosing ADHD in children (refer to Table 5).

**Table 5:** Univariable and multivariable Cox regression analyzes, the influence of demographic factors on ADHD diagnosis age

Demographic variables	*n* (%)	Univariable Risk Ratio (95% CI); *p*-value	Multivariable Risk Ratio* (95% CI); *p*-value
Girl	33 (18.6)	0.68 (0.46–1.01); *p* = .059	0.57 (0.37–0.86); *p* = .007
Has Siblings	135 (76.3)	0.73 (0.52–1.04); *p* = .083	0.63 (0.44–0.91); *p* = .013
PC Age > 40	80 (45.2)	0.79 (0.59–1.08); *p* = .147	0.76 (0.56–1.04); *p* = .084
PC has formal employment	40 (22.6)	1.31 (0.92–1.88); *p* = .140	1.45 (1.00-2.11); *p* = .048
PC Education level **			
Middle school	63 (35.6)	1.06 (0.69–1.64); *p* = .782	
High School / College	83 (46.9)	0.92 (0.61–1.41); *p* = .722	
Health Insurance ***			
Partial health insurance	105 (59.3)	1.34 (0.92–1.97); *p* = .126	
Total health insurance	36 (20.3)	0.78 (0.49–1.26); *p* = .314	

* Variables with *p* ≤ .15 were included in the multivariable model. A backward-stepwise approach was used to eliminate variables from the model (α = 0.10).

** Comparison level is “Primary school”

*** Comparison level is “None”

## Discussion

In Mexico, the majority of caregiving duties fall on women, who are responsible for 94.4% of all caregiving. Almost all primary caregivers in Mexico are women, and nearly half of them are solely responsible for taking care of their children, providing for their families financially, and nurturing them. Single mothers face many challenges when caring for children with ADHD, which may require consultations, therapies, and educational studies, while being the only financial provider for their families. In such cases, they tend to delegate this responsibility to other women in the family, such as the older sister, grandmother, or aunts of the children.

To provide a better understanding of the challenges faced by mother primary caregivers in Mexico, we interviewed a 13-year-old girl and a 72-year-old grandmother. Both the older sister of the 13-year-old and the 72-year-old grandmother were primarily responsible for the children’s upbringing. The outliers were left to highlight the living and working conditions to which Mexican women who have children with ADHD or other mental health disorders are exposed, as well as the difficulties and barriers they must overcome to obtain a formal diagnosis and learn how to treat the disorder.

The sister of the 13-year-old and the 72-year-old grandmother provided excellent information by satisfactorily answering the entire questionnaire. It is important to note that having someone to take care of the child is crucial to evaluate access to timely diagnosis, especially since almost half of women face parenting and provision alone. Therefore, support from their eldest daughters, even if they are minors or their grandmothers, is vital. Without this support, these children would not have the opportunity to receive care for ADHD or any other problem, such as learning or behavior. It is worth emphasizing that teachers and schools are the ones who perceive the signs and symptoms of ADHD and other difficulties as potential mental health problems, not the primary caregivers.

Otherwise, it is important to discuss whether ADHD can be diagnosed at an early stage. According to Brites and colleagues [23], early diagnosis of ADHD is possible. However, it requires a comprehensive interdisciplinary assessment that involves multiple observers and environmental factors, including their impact on the child, peers, and caregivers. Additionally, the American Academy of Pediatrics [1] suggests that the evaluation and diagnosis of ADHD in children and adolescents should start at age 4, taking into account the core symptoms, and academic or behavioral issues. Consensus has been reached by [1, 3–5] that psychoeducation should be the first step in the standard care for preschoolers, based on good clinical practice and the need for multimodal treatment.

Bölte et al. [10] observed that children aged 0–5 show primary symptoms related to psychomotor functions and fundamental interpersonal interactions that are also linked with externalized symptoms. This observation coincides with the Latin American consensus established by Barragán-Pérez et al. [5], which highlights the importance of observing a child’s behavior in various contexts during the preschool period. Different consensuses from various parts of the world agree on the recommendation of early intervention during the preschool stage. It is essential to consider teachers’ opinions and the child’s behavior in the school context. Moreover, it is crucial to determine whether the school provides support services for children who require care. Rocco et al. [7] reported different age ranges for diagnosing ADHD. While Prasad et al. [24] found that 6.8% of children were diagnosed before or at 4, Pohlabeln et al. [25] reported 7.78%. In Mexico City, our results suggest that only 0.6% of children were diagnosed with ADHD before the age of 4, the median age of diagnosis for boys was 7.33 years, while for girls, it was 8.33 years, both boys and girls experienced a delay of 2 years from the time the problem was perceived (Age of Onset), until they received a diagnosis (Age of Diagnosis), as shown in Table 3.

Access to specialized mental health care is a major concern in Mexico. A single study on ADHD conducted in Mexico provided valuable insight into the community’s need for assistance with symptoms and specific behaviors. Caraveo’s results are in agreement with ours since he also highlights the lack of recognition of ADHD symptoms as potential mental health problems. His findings indicate a lack of awareness regarding the significance of some psychopathological manifestations that occur during childhood and adolescence. It is concerning that the critical symptoms of attention deficit hyperactivity disorder, which are among the most prevalent manifestations and also appear early in life, have not been recognized as reasons for concern or as a cause for minors to seek medical care. Only poor academic performance (a consequence of poor attention but not exclusively related to it) was considered an issue.

Caraveo’s study revealed various symptomatic manifestations, such as restlessness, irritability, nervousness, attention deficit, disobedience, explosiveness, and dependent behavior. All symptoms, except irritability, were reported as frequent behaviors that had persisted for over a year, highlighting those related to the core symptoms of ADHD. Despite the significant presence of these symptoms, parents did not consider that their children required mental health care [19].

Studies have found that Latino parents are less likely to seek mental health services for their children, and if they do, they are more likely to abandon treatment early. These findings have been reported by Flores [26] and Kataoka [27]. Furthermore, research on Latin American ethnic groups has revealed that they are at a higher risk of developing externalizing problems such as ADHD, and are less likely to receive school interventions, as reported by Hough [28]. However, Latin American mothers who have experienced a stressful event in their lives are more likely to identify their sons’ problematic behavior early, according to Arcia and Fernández [29]. Asian Americans have a unique perspective on mental illness due to their complex beliefs. In traditional Eastern culture, mental health is not always considered important and is often seen as being in contrast to the concept of self-control [30]; as a result, many Asian Americans believe that they can maintain their mental health by avoiding negative thoughts, strengthening their willpower, and engaging in positive thinking. This suggests that there may be differences in how mental health is perceived and expressed by Asian Americans, as they often describe their symptoms as physical complaints.

Various ethnicities possess unique cultural traits that could impede their access to timely healthcare, especially regarding conditions such as ADHD due to a lack of information. The review highlights that mental health is often viewed through cultural beliefs and customs, not just in Mexican culture. Therefore, disseminating information to the general public is critical, which will enable healthcare providers to become familiar with ADHD and its impact on children’s families, academics, and social lives. This will encourage them to seek professional care for this common disorder.

Fridman and colleagues [14], utilized The Caregiver Perspective on Pediatric ADHD (CAPPA) survey included caregivers of children/adolescents (6–17years) from ten European countries treated with pharmacotherapy in the previous 6 months. Caregivers reported experiences of obtaining an ADHD diagnosis showed a significant difference between the age of onset and the age of diagnosis of a condition in different populations. For instance, the Dutch sample reported the onset of the condition at 2.25 years, while the highest recorded onset was 7.5 years. In Greece, the earliest age of diagnosis was observed at 6.2 years, while the highest mean age was noted in the Swedish population at 18.1 years, and in Lombardia, Italy, the wait time for completing the diagnostic trial varied from three months to one year among 18 different centers. Our findings in Mexico City, it was found that the age of onset for boys was five years old and for girls, it was six years old. The median age of diagnosis for boys was 7.33 years old, and for girls, it was 8.33 years old. Both boys and girls experienced a delay of two years from the age of onset to the age of diagnosis. It took both genders two years from the age of onset to complete the access pathway to obtain a formal diagnosis in specialized mental health services. Another study on access identified insufficient services and gaps in support from healthcare providers/schools.

Previous research has shown that the prevalence of attention-deficit/hyperactivity disorder (ADHD) varies significantly worldwide, primarily due to differences in diagnostic methodologies [31]. As a result, there is no consensus on the optimal age for diagnosis, leading to considerable variation between the age of onset and the age of diagnosis in different populations. For instance, Bonati [12] found that among eighteen centers in different regions of Lombardia, Italy, the average waiting time for completing the diagnostic process ranged from three months to one year. In Mexico City, our research revealed that the age of onset for boys was five years, whereas for girls, it was six years. The median age of diagnosis in boys was 7.33 years, and for girls, it was 8.33 years. Both boys and girls experienced a delay of two years from the age of onset to the age of diagnosis, as shown in Table 3. Additionally, both genders took two years to complete the access pathway and receive a formal diagnosis in specialized mental health services. Kieling and colleagues [13] also studied mental health services and found that the unmet needs of dyads requiring care are not being met, with insufficient services and gaps in support from healthcare providers and schools.

Early diagnosis of ADHD is often challenging due to its association with various psychiatric and somatic disorders. Therefore, it is crucial to consider different comorbidities during screening to get an accurate diagnosis. Comorbidities associated with the disorder’s inattentive subtype, such as depression and anxiety, are among the most frequent and notable mental health disorders [32–34]. Additionally, these comorbidities tend to persist, especially in girls, who may withdraw from social interactions and have difficulty with intimate relationships [3]. The delays observed in diagnosing girls with ADHD, as shown in Fig. 2; Table 4, indicate that it is more difficult for them to receive a timely diagnosis. Even though, the intervention of teachers allowed the underidentification of girls with ADHD and overidentification of boys with ADHD; thanks to the responses we obtained from the primary caregivers (PC), who were the ones who received the complaints, demands, and discontent from the teachers.

Previous research proposal that teachers overidentify externalizing behaviors and underidentify Internalized behaviours [16, 17], and considering the difficulty as well as the delay that girls accessing to specialized mental health services in adition to limited arrival to the host hospital; and considering that are the teachers who identify the problem, the results are consistent with those of Gershon et al. [18], who suggested that differences in symptomatology could account for the delays between boys and girls. Girls with ADHD typically display less severe scores on hyperactivity, inattention, impulsivity, and externalizing symptoms than boys. However, they tend to have more severe intellectual deficits and internalized symptoms [35]. This evidence may explain why girls are often misdiagnosed with impulse control problems instead of ADHD, predominantly inattentive. Therefore, it is necessary to develop a specific diagnostic instrument for girls, as suggested by Victoria et al. [36].

Another obstacle identified to delay the access to care is the absence of a counter-referral system between mental health services at different levels of care is a significant institutional barrier to early diagnosis. This fragmentation of mental health services has a detrimental effect on the overall performance of the system. To address this issue, we recommend implementing the Integrated Health Services Networks method OPS [37].

This method can help to improve coordination between the different levels and sites of care, reduce infrastructure, and provide hospitals with health services in the least stigmatizing place, especially in psychiatric hospitals. To achieve the highest mental health service integration standards, it is advisable to rely on proven techniques such as the Integrated Health Services Networks method. Chile has successfully employed this method to establish an integrated, equitable, and universal mental health system [37]. Therefore, interventions are needed to improve the academic and social difficulties faced by children with ADHD. Besides psychosocial and educational interventions, there is a need to directly address the associated impairment in social and/or academic functioning, along with symptomatic behaviors such as inattention and hyperactivity/impulsivity. Joint health education work would be much more effective in this regard.

Results of this study have shown that socioeconomic factors can significantly impact the timely diagnosis of ADHD in children. Our inferential statistical analysis has identified three key factors contributing to this delay: the child’s gender, the number of siblings, and the occupation of the primary caregiver (PC). Our findings indicated that girls and children with siblings are less likely to receive a timely diagnosis, while children whose PC has formal employment are more likely to receive a timely diagnosis. These findings are consistent with another study that reported that indicators of socioeconomic disadvantage, such as financial difficulties and single-parent status, can result in fewer sources of family support and fewer work-family strategies used by caregivers with partners [38].

Providing workshops for caregivers and their families would benefit mental health services. These workshops would offer advice and strategies for positive parenting. Unfortunately, such services are scarce in Mexico. Currently, the only available psychoeducational workshop is offered by the Children’s Psychiatric Hospital. The workshop is intended for both parents and teachers, and a group of experts, including psychiatrists, neurologists, psychologists, and social workers, teach attendees about ADHD and how it affects children. They also guide how parents and teachers can support children with the disorder. This lack of information and support services for children, caregivers, and teachers is not unique in Mexico. Other authors have also highlighted the urgent need for these services. This is because children with ADHD may develop antisocial or externalizing disorders in adulthood if they do not receive proper support. Masi [39] has emphasized the importance of providing such services to safeguard the well-being of children with ADHD and their social environment.

It is crucial to acknowledge the role of teachers in providing interventions, as without them, accessing mental health services would be challenging for many students. School-based care models have proven successful in addressing this issue in some parts of the world, according to sources, they also agree that the implementation of mental health services in schools is a necessary measure, these services are often located within the educational premises and are an integral part of the educational process [40, 41]. Schools have a long history of providing support services for children who experience mental health problems.

For instance, Mexico had 3,257 Inclusive Education Units (UDEEIS) in 2014 [42]. These units are installed at various educational levels, from preschool to secondary school, to support students with ADHD and other disabilities. However, the support these students receive from UDEEI staff is limited. In most cases, there is only one person to serve the entire school population, dealing with diverse problems that occur in all children and adolescents. Despite these limitations, the UDEEI staff is often the only help that many minors have to go through their academic and mental health difficulties. Their interventions help individuals with ADHD set goals, plan, initiate, and complete tasks efficiently. These interventions also include developing social skills such as giving positive feedback, negotiating, expressing compliments and opinions, and resisting peer pressure. To achieve these goals, the family and the school must collaborate to create a safe and positive environment promoting cordial interpersonal relationships and good behavior.

Three references were provided to supplement the information on the interventions offered by the Special Education and Inclusive Education Unit (UDEEI). The first reference is the Intervention of the UDEEI within the framework of the New Structure of Basic Education Schools, which aims to ensure that boys, girls, and young people can attend school without discrimination. This challenge is closely related to mental health and ADHD, as children with ADHD often face academic difficulties. The interventions offered by UDEEI include advice, support, and counseling for dyscalculia, dysgraphia, and dyslexia [42]. The second reference is the 2011 Program for Strengthening Special Education and Educational Integration, which reports on the level of coverage of UDEEI in Mexico City. According to the report, UDEEI carried out interventions for 2,499 children with ADHD [43]. 

The third reference was included to explain that the UDEEI units are still in an early stage of development as they were introduced in 2015, and their model is still being implemented. This explains why their interventions are limited and not yet proven to be effective. Therefore, it is essential to strengthen these units by working closely with the Ministry of Health to provide UDEEI professionals with training to handle mild cases of ADHD and establish a referral system to refer severe cases to hospitals [44]. 

Mental health and educational authorities need to collaborate to provide mental health services to children and adolescents effectively. They need to form joint participation groups to work together and avoid the isolated approach that mental health services often take. By linking different efforts, we can ensure better access to these services and prevent delays that may harm children’s health, well-being, and academic performance. Joint education-health programs, similar to those in other countries, can help expand non-specialized diagnostic services, shorten access time, and improve coverage. It is crucial to establish agreements between different sectors to combat the existing challenges and meet the significant need for mental health services for children and adolescents.

During our analysis of care needs, we found that there is a lack of information, support, and advice services for minors having ADHD and other issues related to it, such as learning, behavioral, and other problems. We also noticed the need for mental health services to be an integral part of health services. Due to the lack of mental health infrastructure and fragmentation of services, we suggest using the existing infrastructure of the 3,257 UDEEIS in 2014 [42] to improve access to mental health services and provide more suitable and less stigmatizing environments. To achieve this, we propose a significant shift in the organization of diagnostic services for children and adolescents with school-based care models as the primary approach to address the problem of the ideal location for ADHD diagnosis.

According to the recommendations of the PCs, we suggest that the staff and teachers at UDEEI attend the “Management of ADHD in the Classroom” workshop. This workshop encompasses four essential areas: (1) the fundamentals of ADHD, (2) ADHD throughout one’s life, (3) therapeutic interventions for ADHD, and (4) behavioral interventions for ADHD, which include strategies for teachers in the classroom. These workshops have been offered to teachers at the host hospital for over thirty years. The Secretary of Public Education frequently requests them, arguing that they reduce stress and improve teachers’ relationships with their students.

After reviewing the literature on the subject, it has become clear that there is a need for specific psychoeducational programs to be implemented for Latino families and schoolchildren living in the United States as well as in Latinoamerican countries, México including. These programs should aim to recognize the warning signs and symptoms of ADHD and encourage seeking help. Due to ethnic origin plays a crucial role in the early diagnosis process. In Latin American communities, parents tend to prefer psychological treatment overtaking their child to a psychiatrist, which may explain why they don’t often seek out specialty clinics as their first option for care [41].

### Limitations of the study

The study had some important limitations. Firstly, the sample population only included participants from the Host Hospital. This means that the diversity of the sample was limited to this specific group. However, despite this limitation, the study provided an initial exploration that sheds light on the access pathway of children with ADHD and their caregivers. It highlights how they navigate from diagnosis to eventual treatment.

The study was unable to assess the effects of delays caused by insufficient data on different aspects, such as the availability of treatment, satisfaction with care, and the effort made by healthcare providers to help patients diagnosed at different ages. Nevertheless, previous research emphasizes the importance of early diagnosis in enabling more efficient psychoeducational interventions.

It is pertinent to note that the sample size of girls in the study was limited, which is a common characteristic among this population. It is also crucial to acknowledge that girls tend to exhibit more internalized symptoms, which are often less noticeable than the externalized symptoms usually observed in boys. This gender difference may have an impact on the overall understanding and representation of ADHD symptoms within the studied sample.

## Conclusions

Access to specialized diagnostic services for ADHD in Mexico City is significantly delayed for some children and adolescents, particularly girls. This delay suggests that these individuals face great difficulties in getting a timely diagnosis. The delay can have adverse effects on patients, including comorbidities, and prevent them from reaching their full potential. It is urgent to reorganize diagnostic and treatment services by beginning with the Pathway Access from schools since they are the teachers who perceive core symptoms as potential mental health problems; this strategy can reduce delays and prevent future problems. These findings provide strategies to develop a public policy to improve the availability and accessibility of community mental health services.

Therefore, promoting professional training for community workers and regulating professional practice in private settings is crucial to address this issue. Children with ADHD require support to be able to set goals and plans, initiate and carry out tasks efficiently, give positive criticism, negotiate, express a compliment, resolve a conflict peacefully, offer comfort, express their opinion, and resist group pressures, among other social skills. It is vital to understand that the family and the school must jointly promote a safe and positive framework for coexistence and a climate of cordial interpersonal relationships that favor their behaviors and social skills. The health sector must recognize schools, specifically UDEEIS, as providers of these necessary interventions. Mental Health Units in schools are responsive and can play an essential role in the early diagnosis of ADHD.

ADHD can have a significant impact on the lives of children and adolescents, including their families, schools, and social environment. However, many people in the Mexican population, including some teachers and health and mental health professionals, lack sufficient knowledge about this condition. Therefore, it is essential to provide psychoeducational workshops for caregivers and teachers in schools, and to train public health and mental health professionals in the public and private sectors to provide effective care and timely access to diagnosis. Identifying ADHD in primary schools is an opportunity to develop a mental health policy to improve access to timely diagnosis and establish care and support networks. Teachers can play a crucial role in the referral process as they observe children daily and can recognize the academic difficulties that ADHD can cause.

Our rresults suggest that sociodemographic factors affect the delay in diagnosis, including the gender of the child, the number of siblings, and the occupation of the primary caregiver. Unfortunately, these variables are difficult to modify. However, Mental Heath Units inserts at schools (UDEEIS) have been identified as a helpful facilitator in identifing an early diagnosis, as cited in previous research. Therefore, it would be beneficial for the school community to identify the population at a higher risk for delayed diagnosis to provide better support. The study of mental health care system in Mexico, particularly for young children is quite challenging to access mental health services because only 544 outpatient facilities are available, catering to just 310 users per 100,000 inhabitants. Psychiatric hospitals are even fewer, serving only 47 users per 100,000, and there is only one Children’s Psychiatric Hospital in the country. Early intervention is crucial for effective treatment, but unfortunately, there is an inadequate distribution of mental health professionals.

To improve mental healthcare services in Mexico, we propose increasing the availability of services without incurring additional infrastructure costs. Rather than creating new infrastructure, we recommend utilizing existing infrastructure by coordinating services and implementing a referral system between primary, secondary, and tertiary care levels. This includes utilizing the 3,257 UDEEIS units of the Secretary of Public Education that are integrated into public schools in CDMX and currently offering interventions without any connection to the mental health sector [9]. By utilizing the skills of teachers and UDEEIS to detect ADHD and other mental health problems, we can ensure that almost all children receive the necessary attention. Depending on the severity of the problem, we suggest establishing networks at various levels, starting from UDEEI.

Establish strong connections between schools and primary care clinics to enhance access to preventative care and promote a proactive approach towards mental health issues in Mexico City. The Education-Health network can leverage the Medical Specialty Units in Primary Care Centers for Addictions [UNEME CAPA] located in all sixteen mayoralties and the seventy-nine Mental Health Modules and Primary Care Centers for Addictions situated in Health Centers [45]. Unfortunately, these primary care clinics are not linked to schools, resulting in a gap between those who diagnose and treat ADHD and other mental health issues. To bridge this gap, we recommend implementing the alternative Pathway Access approach outlined in this study. This pathway would prioritize early intervention and prevention by encouraging interdisciplinary collaboration and coordination between different levels of care through Public Health Policies. Such initiatives can improve access to mental health support services in Mexico City.

### Key recommendations

- Consider implementing a mental health literacy program in schools that focuses on educating the general population, including parents, teachers, students, and administrative staff. This program should provide information about the signs and symptoms of mental health issues in minors, with a special emphasis on early detection and intervention. The goal is to raise awareness about the importance of recognizing and addressing mental health concerns at an early age.
- It is absolutely crucial to acknowledge and prioritize interventions related to mental health issues in the education sector, utilizing UDEEIS as a powerful tool for this purpose.
- An alternate Access Pathway was identified, requiring intersectoral agreements with the education sector.
- Immediate and drastic reorganization of diagnostic services is imperative to prevent the severe consequences of ADHD and other mental health disorders.
  - Include the optimal age to diagnose ADHD (≤ 4 years of age) in criteria diagnostic. Include the optimal age for ADHD diagnosis (≤ 4 years) in DSM 5 and CIE 10 criteria.
  - Implement a referral and counter-referral system, including UDEEIS, to identify and refer on time and with greater effectiveness.
  - Guarantee training at the 1st level to deal with minor cases and refer severe cases to the third level.
  - Test the effectiveness of instruments to diagnose girls and prevent their unequal access.

## Data Availability

Data can be made available from the corresponding author on reasonable request.

## Abbreviations

ADHD: Attention-deficit/hyperactivity disorder
AcceDa Survey: Acceptability, Accessibility, Availability, and Approach
PC: Primary caregiver
MHP: Mental health professional
HH: Host hospital
